# The Atlanta Society of Medicine

**Published:** 1889-03

**Authors:** 


THE ATLANTA SOCIETY OF MEDICINE is in a flourishing condition. The following are the officers for the present year: Dr. H. P. Cooper, Pres.; Dr. Wm. Perrin Nicolson, Vice Pres.; Dr. E. Van Goidtsnoven, Treas.; Dr N. O. Harris, Corresponding Sec'ty. and Librarian. The Reporters are Drs. E. V. Joye, Frank O. Stockton and W. S. Kendrick.

